# Neurotensin is increased in serum of young children with autistic disorder

**DOI:** 10.1186/1742-2094-7-48

**Published:** 2010-08-23

**Authors:** Asimenia Angelidou, Konstantinos Francis, Magdalini Vasiadi, Konstantinos-Dionysios Alysandratos, Bodi Zhang, Athanasios Theoharides, Lefteris Lykouras, Kyriaki Sideri, Dimitrios Kalogeromitros, Theoharis C Theoharides

**Affiliations:** 1 Laboratory of Molecular Immunopharmacology and Drug Discovery, Department of Pharmacology & Experimental Therapeutics, Tufts University School of Medicine, Boston, MA, USA; 2 Second Department of Psychiatry, Attikon General Hospital, University of Athens Medical School, Athens, Greece; 3 Allergy Clinical Research Center, Allergy Section, Attikon General Hospital, University of Athens Medical School, Athens, Greece; 4 Department of Biochemistry, Tufts University School of Medicine, Boston, MA, USA; 5 Institute of Social Health Insurance (IKA), Thessaloniki, Greece; 6 Department of Internal Medicine, Tufts University School of Medicine and Tufts Medical Center, Boston, MA, USA; 7 Department of Psychiatry, Tufts University School of Medicine and Tufts Medical Center, Boston, MA, USA

## Abstract

Autism spectrum disorders (ASD) are a group of pervasive neurodevelopmental disorders diagnosed in early childhood. They are associated with a set of "core symptoms" that include disabilities in social interaction skills, verbal and non-verbal communication, as well as repetitive and stereotypic behaviors. There is no definite pathogenetic mechanism or diagnostic tests. Many children with ASD also have "allergic-like" symptoms, but test negative implying mast cell activation by non-allergic triggers. We measured by Milliplex arrays serum levels of 3 neuropeptides that could stimulate mast cells in children with autistic disorder (n = 19; 16 males and 3 females; mean age 3.0 ± 0.4 years) and healthy, unrelated controls (n = 16; 13 males and 3 females; mean age 3 ± 1.2 years). Only neurotensin (NT) was significantly increased from 60.5 ± 6.0 pg/ml in controls to 105.6 ± 12.4 pg/ml in autistic disorder (p = 0.004). There was no statistically significant difference in the serum levels of β-endorphin or substance P (SP). NT could stimulate immune cells, especially mast cells, and/or have direct effects on brain inflammation and ASD.

## Background

Autism spectrum disorders (ASD) are a group of neurodevelopmental disorders that include autistic disorder, Asperger's disorder, and pervasive developmental disorder-not otherwise specified (PDD-NOS) [1,2]. These are diagnosed in early childhood and are characterized by a set of "core symptoms" that include various degrees of disability in social interaction skills, verbal and non-verbal communication, as well as limited interest in activities often associated with repetitive and stereotypic behaviors [1,2]. The diagnosis of ASD has increased during the last decade to about 1/100 children [3,4]. However, there is neither distinct pathogenesis, nor reliable biomarkers [5].

A number of papers have suggested that ASD may be associated with some immune dysfunction in the patient [6] or the mother during gestation [7]. More recently, evidence has been reviewed suggesting that ASD may have a neuroimmune component involving non-allergic activation of mast cells by triggers such as neuropeptides [8]. However, few studies have measured levels of neuropeptides in autism. Most of the investigations have focused on oxytocin and vasopressin [9].

In one paper, archived neonatal blood was analyzed with immunoaffinity chromatography and serum levels of vasoactive intestinal peptide (VIP) and calcitonin-gene related peptide (CGRP) were higher in children with ASD (n = 69) and those with mental retardation without ASD (n = 60); in contrast, levels of substance P (SP) and nerve growth factor (NGF) were similar to those of controls [10]. However, a subsequent study by the same author using Luminex immunoaffinity arrays showed no difference in any of these molecules between autistic subjects and controls [11]. Another study showed significantly elevated levels of β-endorphin in the cerebrospinal fluid (CSF) of children with infantile autism (n = 9), but serum levels were not measured [12].

We investigated serum levels of β-endorphin, neurotensin (NT) and SP, all of which are present both in the brain and the gut and are known mast cell triggers [13]. Here we report that NT is the only neuropeptide significantly elevated in the serum of children with autistic disorder as compared to unrelated age-matched, normally developing, control children.

## Methods

### Study population

Patients came from the Second Department of Psychiatry at Attikon General Hospital, University of Athens Medical School (Athens, Greece), an NIH-approved site for biological samples under a collaboration agreement between Athens and Tufts Universities.

### Patient assessment

All children were assessed by trained ASD clinicians. Parents signed an appropriate consent form according to the Helsinki principles. The ADI-R and ADOS-G scales were used as they have also been validated in the Greek population [14]. The Childhood Autism Rating Scale (CARS) was completed as a further measurement of the severity of ASD. A test for Fragile × syndrome was ordered and the subjects were screened with Wood's Lamp for tuberous sclerosis.

The inclusion and exclusion criteria for the study were as follows:

### Inclusion criteria

1. Must meet ICD-10 criteria for autistic disorder.

### Exclusion criteria

1. Any medical condition likely to be etiological for ASD (e.g. Rett syndrome, focal epilepsy).

2. Any neurologic disorder involving pathology above the brain stem, other than uncomplicated non-focal epilepsy.

3. Contemporaneous evidence, or unequivocal retrospective evidence, of probable neonatal brain damage.

4. Any genetic syndrome involving the CNS, even if the link with autism is uncertain.

5. Clinically significant visual or auditory impairment, even after correction.

6. Any circumstances that might possibly account for the picture of autism (e.g. severe nutritional or psychological deprivation).

7. Active treatment with pharmacological or other agents.

8. Mastocytosis (including urticaria pigmentosa).

9. History of upper airway diseases.

10. History of inflammatory diseases.

11. History of allergies.

There were no other apparent clinical differences (e.g. gastrointestinal problems) that may have allowed separation of the autistic patients in subgroups. The ASD sample consisted of 19 children with autistic disorder. The controls were normally developing, healthy children, unrelated to the autistic subjects, without any of the exclusion criteria (n = 16; 13 males and 3 females; mean age 3 years); these subjects were seen for routine health visits at the Pediatric Department of the Institute of Social Health Insurance, Greece. There were no identifiers except for age and sex.

### Objective measurements

Blood was obtained in the morning at least 2 hours after breakfast to minimize any diurnal or postprandial effects. Serum from patients and controls was aliquoted and frozen at -80°C until assayed. All samples were labeled only with a code number, as well as the age and sex of the respective subject. Samples were analyzed for neuropeptides and cytokines using Milliplex MAP, based on the Luminex xMAP technology by Millipore (Billerica, MA).

### Statistical analysis

The results for serum neuropeptide levels are presented as scattergrams, with the horizontal lines indicating the means, in order to appreciate the distribution of the values. The ASD group was compared to the controls using unpaired, unequal, 2-tailed, Student's *t*-test, as well as the non-parametric Mann-Whitney *U *test. Significance of comparisons between healthy subjects and subjects with ASD is denoted by p < 0.05.

## Results

The ASD group consisted of 19 patients (16 males and 3 females; mean age: 3.0 ± 0.4 years; range: 2.5-3.5 years) all with a diagnosis of autistic disorder. The control group consisted of 16 subjects (13 males and 3 females; mean age: 3 ± 1.2 years; range: 2-5.5 years). These were healthy, normally developing subjects, unrelated to the ASD patients.

**Figure 1:**
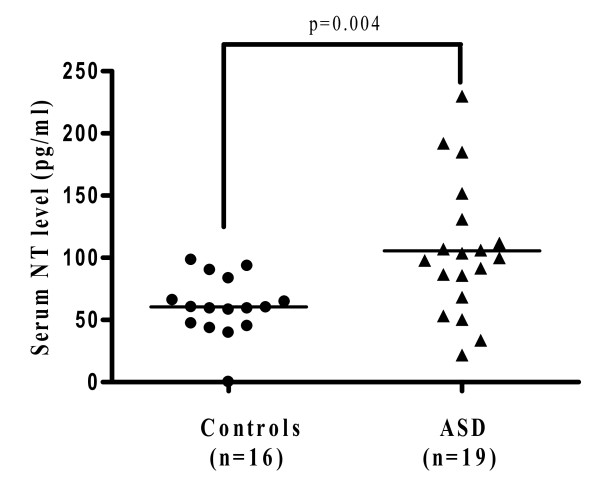
**Serum levels of neurotensin in ASD patients (n = 19; 16 males and 3 females; mean age: 3 ± 0.4 years; range: 2.5-3.5 years) and controls (n = 16; 13 males and 3 females; mean age: 3 ± 1.2 years; range: 2-5.5 years)**. Serum was analyzed using Milliplex MAP, based on the Luminex xMAP technology by Millipore. The horizontal lines indicate the means.

**Figure 2:**
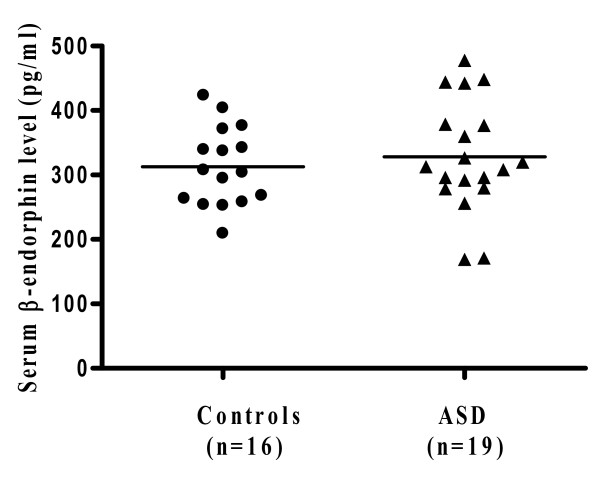
**Serum levels of β-endorphin in ASD patients (n = 19; 16 males and 3 females; mean age: 3 ± 0.4 years; range: 2.5-3.5 years) and controls (n = 16; 13 males and 3 females; mean age: 3 ± 1.2 years; range: 2-5.5 years)**. Serum was analyzed using Milliplex MAP, based on the Luminex xMAP technology by Millipore. The horizontal lines indicate the means.

**Figure 3:**
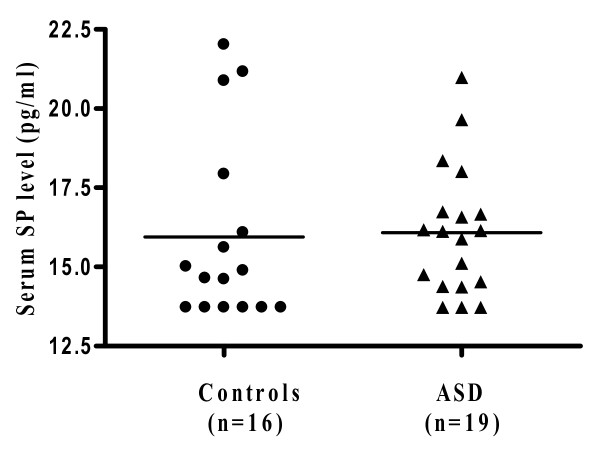
**Serum levels of substance P in ASD patients (n = 19; 16 males and 3 females; mean age: 3 ± 0.4 years; range: 2.5-3.5 years) and controls (n = 16; 13 males and 3 females; mean age: 3 ± 1.2 years; range: 2-5.5 years)**. Serum was analyzed using Milliplex MAP, based on the Luminex xMAP technology by Millipore. The horizontal lines indicate the means.

Serum was analyzed for the neuropeptides β-endorphin, NT and SP. Only NT was significantly elevated (Figure 1) compared to controls (p = 0.004). Specifically, the serum NT level in the subjects with autistic disorder was 105.6 ± 12.4 pg/ml, as compared to 60.5 ± 6.0 pg/ml in the controls. There was no statistical difference in the serum levels of β-endorphin in autistic subjects (328.0 ± 19.7 pg/ml) compared to the controls (312.8 ± 15.3 pg/ml) (Figure 2). Serum levels of SP did not differ between the 2 groups either (16.1 ± 0.5 in ASD compared to 16.0 ± 0.7 in the controls) (Figure 3).

We also investigated serum levels of cytokines usually associated with mast cells (IL-4, IL-6, IL-10, IL-13, TNF), but there was no statistically significant difference (results not shown).

## Discussion

Our present results indicate that NT is elevated in the serum of young children with autistic disorder, as compared to unrelated, normally developing controls. All subjects were selected to exclude any allergic, inflammatory or neurologic diseases in order to avoid any confounding factors. The distribution of NT values in the ASD subjects suggests there may be two subgroups. However, nothing in the history or physical examination allowed such subdivision. SP was not elevated as also previously reported [10,11]; β-endorphin was also not elevated, even though it had been reported to be increased in the CSF of a small group of children (n = 9) with infantile autism [12].

This is the first study to report measurements of NT in the serum of human subjects with Luminex microbead arrays. No *physiological levels *of NT have been established for any disease state yet. However, one study reported plasma NT levels measured by RIA in healthy neonates to be 59.1 ± 23.9 pg/ml [15]. Another study evaluated the role of NT in growth regulation in subjects of different ages and reported that plasma levels gradually declined with increasing age with plasma levels in prepubertal children being 22.2 ± 4.8 pg/ml [16].

NT is a peptide originally isolated from the brain [17]. It is present also in the gastrointestinal tract, where it can induce intestinal inflammation [18]. NT can stimulate lymphocyte proliferation [19], activate T cells [20], and enhance IL-1 production from macrophages [21]. NT is also a potent trigger of mast cells [22]. These effects are relevant to the findings that many children with ASD also present with gastrointestinal and "allergic-like" symptoms [23]. In particular, mast cells appear to be activated in ASD as suggested by more food allergies [24] and increased atopic symptoms in Asperger patients [25]. Such "allergic symptoms" often occur in the absence of elevated serum IgE or positive skin prick tests [26], suggesting mast cell activation by non-immune triggers [13]. Moreover, a preliminary report indicated that ASD is 10-times more frequent in mastocytosis patients (1/10 children) [27] than the general population (1/100 children) [4]. Mastocytosis is a rare disorder, which presents with skin reactions, food allergies or food intolerance, diarrhea, anxiety [28], but also lack of concentration ("brain fog"), and hyperactivity [29].

NT could be released from the brain, the intestines or dorsal root ganglia and could act together with environmental triggers such as mercury [30] or corticotropin-releasing hormone (CRH), secreted under stress, to stimulate mast cells and lead to neurogenic inflammation [31]. The possible release of NT under stress may be relevant to the finding of higher incidence of prenatal stressors in mothers of children with ASD [32]. The present study did not attempt to correlate serum NT to levels of stress. Future studies could employ the Hamilton Anxiety Scale or the Spielberger's State-Trait-Anxiety Inventory, as well as measure serum levels of cortisol, adrenocorticotropic hormone (ACTH) and CRH. It is interesting that mast cells can degrade NT [33,34]. This action suggests that mast cells have developed a rapid mechanism for limiting stimulation by NT that implies an important pathophysiological role.

Mast cells are involved in both innate and acquired immunity [35], as well as in inflammation [13]. Moreover, mast cells can release some mediators "selectively", without concomitant secretion of either one of their "flagship" molecules histamine or tryptase [36], making serum measurement of these mediators as biomarkers irrelevant. Moreover, histamine is metabolized rapidly, while tryptase is elevated only in anaphylaxis and mastocytosis, conditions that were part of the exclusion criteria. It is not apparent at present which mast cell mediators are secreted in response to NT. Our preliminary findings on Luminex measurements of IL-4, IL-5, IL-6, IL-8, IL-13 and tumor necrosis factor (TNF) did not show any significant difference (results not shown). Even though IL-6 expression was elevated in the brains of deceased ASD patients [37], and it was detected at low levels in the CSF in subjects with autism (n = 12), it was not significantly elevated in the serum of autistic subjects (n = 35) compared to control subjects with other neurologic disorders (n = 21) [38]. Elsewhere, there was only a trend towards increased production of IL-6 and TNF-α in *whole blood *of autistic children as compared to normal controls [39]. Another study reported that TNF-α levels in CSF of patients with ASD (n = 10) were significantly higher than concurrent serum levels [40]. TNF is uniquely stored in mast cell granules [41], and brain mast cells were reported to secrete TNF [42]. It may well be that NT levels are sufficient to activate only brain and gut mast cells, thus not significantly raising systemic levels. Alternatively, mast cells may release additional mediators that have not been identified so far.

The present results indicate that NT is increased in young children with autistic disorder and could participate in altered innate immunity and brain inflammation.

## Competing interests

The authors declare that they have no competing interests. TCT is the inventor of US patent application 12/534,571 on "Methods of diagnosing and treating Autism Spectrum Disorders and compositions for same", which has been assigned to Theta Biomedical Consulting and Development Co., Inc. (MA).

## Authors' contributions

AA, analyzed the results and helped write the paper. KF and KS collected all the autistic samples and reviewed the results. MV and KDA helped analyze the results and write the paper. BZ helped analyze the results. AT provided all the normal controls. LL and DK supervised the collection of the human samples. TCT designed the study, organized the collection of human samples, supervised the analysis of the results and wrote the paper. All authors have read and approved the final version of the manuscript.
